# “Because Work Time Is Life Time” – Employees’ Perceptions of Individual Overemployment, Its Causes and Its Consequences

**DOI:** 10.3389/fpsyg.2019.01920

**Published:** 2019-08-16

**Authors:** Julia Hiemer, Maike Andresen

**Affiliations:** Chair of Human Resource Management and Organisational Behaviour, Department of Social Sciences, Economics, and Business Administration, University of Bamberg, Bamberg, Germany

**Keywords:** overemployment, work time, work time preferences/desires/intentions, work hours, Grounded Theory

## Abstract

Many employees would prefer to reduce work time and can be defined as overemployed. However, the concept of overemployment is poorly understood. The purpose of this article is to define overemployment from employees’ point of view, to explain why people work more than they prefer, and to understand the individual consequences it has. We investigate 26 overemployed employees using a Grounded Theory approach. We find that overemployment is a four-dimensional experience consisting of work time length, work time competition (with time outside work), work time distribution on tasks, and work density. A self-reinforcing circle of personal and situational drivers seems to explain the persistence of overemployment. Regarding the psychosocial consequences of overemployment, our findings show large variations, whereby work time sovereignty seems to play a moderating role. This study provides a multidimensional framework of overemployment that provides a basis for understanding employees’ perceptions and behavior regarding overemployment and for deriving appropriate actions to reduce overemployment.

## Introduction

“Work time actually is life time, too. I don’t want to spend my lifetime only at work.” (7)^1^

Long working hours and blurred boundaries between work life and private life are common among professionals (Eurofound, 2017; Ross et al., 2017). For employees, there may be acceptable reasons for working long days, such as expected positive career outcomes, e.g., in terms of salary (Spurk and Abele, 2011) or intrinsic rewards (Brett and Stroh, 2003). In addition, companies may encourage employees to work extra hours: they are an indicator of employee performance (Kmec et al., 2014) and lead to sought-after lower relative labor costs (Boulin et al., 2006).

The phenomenon of overemployment must be distinguished from long working hours *per se*. Overemployment is usually defined as a state in which an employee, working full-time or part-time, would prefer to work less (work fewer hours) than is currently the case (e.g., Golden and Gebreselassie, 2007; Wooden et al., 2009; Golden, 2014), while accepting reduced earnings in consequence (van Echtelt et al., 2006). Overemployment is a widespread phenomenon. In Europe, around 30% of all employees would prefer to work fewer hours (albeit with strong variations between countries; Eurofound, 2017, data based on 35 European countries).

While literature finds no direct effect of long work hours *per se* on either physical or mental well-being (Ganster et al., 2016), this is not true for overemployment (Wooden et al., 2009; Angrave and Charlwood, 2015). Therefore, we regard the subjective experience of overemployment as more important than the objective hours worked. Prior research predominantly finds that overemployment is detrimental to individuals’ well-being: it is negatively related to job satisfaction (Wooden et al., 2009; Wunder and Heineck, 2013; Angrave and Charlwood, 2015), life satisfaction (Wooden et al., 2009) and psychological well-being (Angrave and Charlwood, 2015). Job dissatisfaction negatively correlates to performance and retention (Judge et al., 2001). These results highlight the importance of studying overemployment, rather than long work hours, for the individual and for organizations.

Managing overemployment from the individual to the organizational level requires solid knowledge about the phenomenon. However, the understanding of overemployment is still in its infancy and this has an impact on study results. Regarding the measurement of overemployment, many previous studies focus solely on economic aspects, viewing overemployment as a trade-off problem between money and leisure (e.g., Böheim and Taylor, 2003). This represents a simplified view of peoples’ motivation to work, as there can be many other reasons to work beyond earning an income (e.g., see Conklin, 2011 study on calling). In addition, previous overemployment studies rely on working time preference data from large-scale surveys (Golden and Gebreselassie, 2007). A common method here is asking employees about their current hours and then asking if they would prefer to work fewer weekly hours. These data, however, show strong deficiencies, as answers to working time preference questions are not only instable, but also prone to wording effects, as Holst and Bringmann (2016) demonstrate. Therefore, it is crucial not only to analyze overemployment by asking employees about their actual and preferred number of working hours, but also to ask how they *perceive* overemployment. Campbell and van Wanrooy (2013) undertook an important step toward this. They interviewed overemployed persons to gain more comprehensive insights into their experiences. However, there are some limitations to their study. The first of these is related to sample selection: Campbell and van Wanrooy (2013) only included full-time employees working five or more extra unpaid hours, whereas overemployment, by definition, is a subjective phenomenon, i.e., people can feel overemployed regardless of how much or how little they actually work. Thus, leaving out the subjective estimation of overemployment in the selection of participants may limit research results. The second limitation refers to Campbell and van Wanrooy’s (2013) research questions: they mainly focused on the ambivalence and usability of work hours preference questions. We build on this research, but go a step beyond it: where Campbell and van Wanrooy (2013) still rely on preference questions to interpret people’s answers, we apply a Grounded Theory approach and try not to impose prior concepts on participants, but to focus on their own construction of overemployment (Gehman et al., 2018). In addition, our research questions go beyond Campbell and van Wanrooy’s (2013) study by including the causes of overemployment. Researchers have also found that overemployment tends to persist for long periods of time (Reynolds and Aletraris, 2010) and we aim to gain insights into what contributes to this persistence. Moreover, we investigate consequences of overemployment as perceived by employees. Thus, we address the following questions:

(1) How is “overemployment” defined from the perspective of employees?
(2) What are the perceived causes of overemployment, and what contributes to its persistence?
(3) What are the consequences of overemployment for individuals?

Answering these questions is important for theory and practice. Our contribution is threefold: First, a better understanding of the overemployment concept serves as a basis to develop a better measurement of overemployment. Measuring overemployment is also important for practice, e.g., for diagnosing overemployment before defining actions to improve work time satisfaction. Second, our Grounded Theory approach allows detailed insights into the causes and mechanisms of the persistence of overemployment that serve to identify levers for managing overemployment. Finally, our Grounded Theory analysis leads to propositions on the causes and consequences of overemployment that may guide future research.

Current research on overemployment has focused mainly on its measurement and on the identification of causes and consequences. However, a huge variety of approaches can be found, hampering the comparability of findings.

The definition of overemployment and its operationalizations diverge widely (Fagan, 2001; Wooden et al., 2009), and in consequence, reported overemployment rates vary drastically between studies (Holst and Bringmann, 2016). One major difference between definitions is whether they explicitly include the assumption that income is reduced when reducing hours (for a more detailed overview of variations in the definition and measurement of overemployment, see Golden and Gebreselassie, 2007). In line with other researchers (e.g., Brown and Sessions, 2001; Golden and Gebreselassie, 2007; Abrahamsen, 2010), we do not see a reduction of current income as a necessary facet of overemployment. As not all employees are paid by the hour, reducing work time may not necessarily reduce incomes. Income reduction is consequently not always included in overemployment definitions (e.g., Golden and Gebreselassie, 2007). Moreover, people may not only consider short-term income reductions, but also long-term consequences of reducing their hours (e.g., in terms of their career or individual development), and they may take such consequences into account even when they are not expressly asked to consider them. Overemployment measures also differ with regard to quantification, i.e., whether people are asked for their exact hours preferences (van Echtelt et al., 2006) or about whether they want a reduction in their hours that is not precisely quantified (e.g., Abrahamsen, 2010). In line with research that shows that it is too difficult for employees to indicate their exact weekly working hours preferences precisely (Campbell and van Wanrooy, 2013), we refrain from asking study participants to indicate their exact hours preferences (Gioia et al., 2013). We apply a working definition of overemployment that only includes core aspects of overemployment to give ourselves space to listen to what our informants are telling us without being overly biased by prior research (Gioia et al., 2013). Thus, we start with a preliminary definition of overemployment as an *imbalance between preferred and actual working time*, where *actual working time exceeds preferred time*.

Moreover, as argued above, overemployment as a *subjective phenomenon* is independent of long working hours. Overemployment can be differentiated from long work hours *per se*, as the latter refers to work hours that exceed the standard full-time work week, but without implying a preference for working fewer hours (Beckers, 2008). Prior studies have found that not only people working longer, but also people working fewer than 40 h per week often wish to reduce their work hours (e.g., Reynolds, 2003). The perception of overemployment is distinct from job dissatisfaction, which generally refers to whether a job is enjoyable or not (Warr, 2007). Not all people wanting to reduce their work hours are necessarily dissatisfied with their jobs (e.g., Brett and Stroh, 2003; Reynolds and Aletraris, 2007). Reynolds and Aletraris (2007) found that individuals are not only less likely to desire a decrease in work hours as their job satisfaction increases, but also that people do not want to spend more hours even on jobs they find satisfying. Spending too much time at work may make a job less satisfying (Reynolds and Aletraris, 2007). Long work hours and overemployment are thus related but conceptually distinct phenomena. To conclude, we see the need to define overemployment from the employee perspective (research question 1) and to provide a basis for the improvement of its measurement.

Apart from the definition and measurement of overemployment, the causes of overemployment have been investigated in prior research. However, results regarding the causes of overemployment have been inconclusive so far (Reynolds and Aletraris, 2010). In the economic model of labor supply, it is assumed that overemployment is a result of labor market demand, i.e., firms offer fixed hours-wage packages, restricting employees in their choice of working hours (e.g., Altonji and Paxson, 1988; Böheim and Taylor, 2004). Employers have an incentive to hire employees only for a substantial number of hours. Given the limited availability of positions, the chances of being appointed to a position with exactly an individual’s preferred working hours are rather limited (Altonji and Paxson, 1988). In contrast to this traditional explanation, Landers et al. (1996) and Eastman (1998) focus more on competition among employees and the jockeying for position that leads employees to work long hours. van Echtelt et al. (2006) concentrate on aspects of post-Fordist work organization (e.g., high autonomy, project work, deadlines, competition) that cause overemployment. Other authors have focused on finding demographic characteristics that correlate with overemployment, e.g., having children (Reynolds and Johnson, 2012), being married, or possessing a higher level of education (Golden and Gebreselassie, 2007). Each of these studies focuses on individual aspects; none of them offer an integrated view that sufficiently explains what causes overemployment and what leads to its persistence. The present study therefore sets out to capture an integrated view of employees’ perceptions of the causes of overemployment (research question 2).

Regarding the consequences of overemployment (research question 3), well-being and job satisfaction are the variables that have been most comprehensively investigated. Angrave and Charlwood (2015) adopt a person-environment fit framework and hypothesize that it is not the length of the working week in absolute terms, but the fit between actual and preferred working hours that affects the subjective well-being of workers. Earlier studies, however, only partly confirm this hypothesis. Regarding well-being, Wooden et al. (2009) as well as Angrave and Charlwood (2015) found significant effects of overemployment on life satisfaction, whereas Friedland and Price (2003) and Wunder and Heineck (2013) found no such effects. Bell et al. (2011) found negative effects of overemployment on health satisfaction and self-assessed health. Overemployed people in Friedland and Price (2003), study by contrast, reported a higher prevalence of chronic disease, but not lower health satisfaction, and, surprisingly, lower depressive symptoms. Respondents in Angrave and Charlwood’s (2015) study showed lower psychological well-being. This inconsistency in findings is probably due to individual perceptions having been neglected to some degree in the conceptualization of these studies and, following on from this, to a degree of inaccuracy in the measurement of overemployment (Campbell and van Wanrooy, 2013; Holst and Bringmann, 2016). For this reason, patterns of overemployment consequences are also included in our investigation (research question 3).

## Materials and Methods

### Research Strategy

We employed a qualitative, Grounded Theory research approach we considered to be the most appropriate option given the limited development of the overemployment concept (Edmondson and McManus, 2007); most prior studies have relied on quantitative overemployment data. However, these studies neither analyzed what overemployment means from the perspective of those affected nor explored how people affected by overemployment explain its causes. Although there is a significant body of literature on overemployment, the phenomenon remains paradoxical: why do so many people wish they had less work time, yet not reduce their hours? As Edmondson and McManus (2007) suggest, inductive research, e.g., Grounded Theory, is a perfectly fitting method here for “digging into a paradoxon” (Edmondson and McManus, 2007, p. 1162).

For developing a better understanding of the concept of overemployment as well as its consequences, we followed the Grounded Theory approach established by Gioia (see e.g., Gioia et al., 2013; Murphy et al., 2017). According to Gioia et al. (2013), much effort is often invested in concept elaboration, but little in the “more important work of concept development” (Gioia et al., 2013, p. 16). This is also the case for the construct of overemployment: although a considerable body of research on overemployment exists (e.g., Golden and Gebreselassie, 2007; Reynolds and Aletraris, 2010), the construct is still conceptually unclear and attempts to measure overemployment are distorted by ambivalence (Campbell and van Wanrooy, 2013; Holst and Bringmann, 2016). In addition, a universally accepted theory of overemployment is lacking, and much research is guided by established theories from other areas, e.g., person-environment fit theory (Angrave and Charlwood, 2015) or self-discrepancy theory (Odle-Dusseau et al., 2012). The Gioia Grounded Theory approach assumes that organizational phenomena are socially constructed by “people [who] know what they are trying to do and can explain their thoughts, intentions, and actions” (Gioia et al., 2013, p. 17). Therefore, we interviewed overemployed people and tried to stay close to their experiences when interpreting the data. We also followed the principle of starting with a preconceived structured interview guide that was tailored to our research question (see Appendix A), but flexible enough to change as research progressed (Gioia et al., 2013). In addition, we followed Gioia et al. (2013) by primarily proceeding in a bottom–up fashion and taking care not to allow existing literature to bias our research findings too much.

### Sample and Sampling Strategy

To find people who were currently experiencing overemployment, requests were posted on social networking sites that are popular in Germany (LinkedIn, Xing). Germany was chosen because of its significant proportion of employees reporting overemployment according to long-term data from the German Socio-Economic Panel (e.g., Wunder and Heineck, 2013). decided to only use people working in Germany and not to mix countries, as cultural (e.g., values) and structural (e.g., legal and economic circumstances) aspects differ considerably between countries (Ollier-Malaterre and Foucreault, 2016) and this makes results difficult to compare. To make sure we selected an adequate sample, we explicitly asked for people who “currently experience imbalances between preferred and actual working time, where actual working time exceeds preferred working time.” We chose subjects who classified themselves according to this definition, as we were interested in the subjective experience of overemployment. We did not use a contrasting subsample, i.e., two samples with a relevant contrasting feature; as the phenomenon of overemployment is still vaguely defined, we considered that no reliable criterion variable that could have been used to split the samples would be identifiable (Boyatzis, 1998).

The Grounded Theory approach of theoretical sampling was used here. This means that our sample was not selected to be representative of a group of people, but representative in terms of concepts (Charmaz, 2014). Derived from a maximum variation sampling strategy (Patton, 1990), our approach purposefully sought to interview people with different job and personal circumstances and diverse work time arrangements to ensure a large degree of variability between different cases (see Table 1). Any pattern emerging from that large variation thus captures the core experiences relevant for developing our theory (Patton, 1990). All respondents had gained, at a minimum, a school-leaving certificate qualifying for university entrance, since our interviews required participants with good language skills. Our approach involved an iterative process of simultaneously collecting and analyzing data and seeking new informants based on the information that had been gleaned and deemed important in prior interviews (Glaser and Strauss, 1967; see also Gioia et al., 2010). We continued sampling until theoretical saturation was reached, i.e., interview data ceased to yield any new conceptual themes or insights (Glaser and Strauss, 1967; Charmaz, 2014). Initial interviewing began with employees working in the consulting sector (Interviewees 1–5) and in the banking and finance sector (Interviewees 6–10) because long working hours are common in these sectors (Hewlett and Luce, 2006). From these first interviews, tentative ideas were developed that were examined further by searching for new data that could be used to refine or reject our initial ideas (Charmaz, 2014). We interviewed people with and without children, as well as people in leadership positions (Interviews 6, 19, 20, 21, 24) and people in special situations (Interviewee 13, doing a Ph.D. alongside work, or Interviewee 17, with a very long commute) as these factors might influence perceptions of work hours. This led to a sample of 26 interviewees who described being overemployed (see Table 1).

**TABLE 1:** Interviewees’ basic profiles.

				**Self-reported weekly**
**Interviewee**	**Sex, age^1^**	**Job description (industry)**	**Family Status**	**work hours**
1	M, 33	Marketing consultant (consulting)	Partner, no children	50
2	F, 31	Senior recruiter (consulting)	Married, no children	45
3	F, 32	Senior HR officer (consulting)	Partner, no children	45
4	F, 60	Senior HR officer (consulting)	Married, one child	38
5	M, 52	Consultant (self-employed)	Single, 3 children	60
6	M, 44	Departmental head (banking)	Married, 3 children	50
7	F, 58	Specialist (banking)	Single, two children	42
8	F, 27	Personnel officer (finance)	Partner, no children	45
9	M, 28	Assistant to the CEO (banking)	Partner, no children	50–55
10	M, 30	HR development (banking)	Married, one child	45
11	F, 30	Copywriter (advertising agency)	Partner, no children	45
12	F, 38	Market research specialist (market research company)	Married, one child	50
13	M, 28	Project manager (agency)	Partner, no children	30
14	F, 34	Junior data manager (pharma)	Married, no children	41
15	M, 51	Principal expert software ergonomics (engineering company)	Married, 3 children	40
16	M, 25	IT developer (IT)	Married, one child	52,5
17	F, 33	Commercial clerk (telecommunication)	Married, no children	48
18	F, 29	Online editor (retail)	Partner, no children	41
19	M, 59	Human Resources Director (retail)	Married, two children	45
20	M, 44	Work design specialist (automobile)	Single, no children	44
21	M, 31	Chef (catering company)	Married, two children	55
22	F, 55	Receptionist/Team assistant (media)	Married, two children	40
23	F. 29	Personal assistant to management (food)	Married, no children	40
24	F, 46	Professor (university)	Married, two children	70
25	F, 36	Academic Council (university)	Married, one child	46
26	M, 28	Research Associate (university)	Single, no children	55

*^1^F, female; M, male.*

The average contractual work week was 39 h (two people had no fixed hours but a range of 30–40 h). The average reported actual time worked was 46 h per week (including overtime hours, not including commuting time). Three people had part-time contracts, while all others had full-time contracts. Nineteen people were not paid for overtime, three were partially paid and four were fully paid. The mean commute was 1.5 h per day (range: 0.3–2.5 h). Out of 22 respondents with partners, 14 had partners in full-time employment, four had partners in part-time employment, two had self-employed partners and two had non-employed partners. Employees’ gross income stood at 4,390 Euro per month (range: 1,900–10,000 Euro; *SD* = 2,390).

### Data Collection

Overall, we conducted 26 interviews that lasted 45 min on average. About a week before the interviews, participants provided additional sociodemographic information with the help of a 5-min online questionnaire that served to aid the meaningful interpretation of responses (Corbin and Strauss, 1990). Interviews were conducted via telephone by the first author, recorded and then transcribed. Participants gave written informed consent for research participation as well as for the use of their data in anonymized form in research and publications. In line with Gioia et al.’s (2013) Grounded Theory approach, we used an interview protocol focusing on the research questions. Initially, a general question about the interviewees’ current work time situation was asked, followed by questions about satisfaction with work time, feelings about work time and ideal work time. Later, we posed questions about causes of overemployment (see Appendix A). As our research progressed, we also repeatedly revised the protocol to follow the course of the research (Gioia et al., 2013). We mainly asked open questions (e.g., asking “Tell me about …!”, “Why?”, “How?”, “What?”) to best capture the participant’s own words. By doing so, following the Gioia approach, we treated our interviewees as “knowledgeable agents” and tried not to impose prior theory or concepts on them (Gehman et al., 2018).

### Data Analysis

Each interview was transcribed and analyzed directly after having been conducted. In each step of the analysis outlined below, two coders first independently, i.e., without seeing the judgment of the other observer (Boyatzis, 1998), performed the coding step and met at regular intervals to discuss their individual results and reconcile discrepancies. Following Gioia et al. (2013), we continually revisited the data, engaged in discussions, and reconciled differing interpretations by developing consensual decision rules about how terms were to be coded. Throughout this procedure, the two coders read the interviews multiple times and the codes were revised when considered necessary (Charmaz, 2014). Coding was performed following the Grounded Theory methodology (Gioia et al., 2013; Charmaz, 2014) and applying the ideas of Thematic Analysis. Thematic Analysis is a process for encoding qualitative information that can be used as part of qualitative methodologies like Grounded Theory (Boyatzis, 1998). Throughout the coding process, the two coders developed notes that ensured the codes contained the characteristics of good code according to Thematic Analysis, i.e., the name and definition of the code and a description of indicators for when and when not to use the code including examples (Boyatzis, 1998). Coding was done in four steps (for detailed descriptions, see Appendix C).

Step 1: Open coding. Two coders (the first author and a research assistant) independently began by reading each transcript and generating “*in vivo*” codes, i.e., meaningful terms used by informants or reflecting the level of meaning and the language of informants (Strauss and Corbin, 1998; Gioia et al., 2010). Some *in vivo* codes are highlighted in Appendix B.

Step 2: First-order categories. The same two coders independently grouped all *in vivo* codes into higher-level concepts based on underlying similarities. Examples of first-order categories are embedded in the Results section and Table 2.

**TABLE 2:** Overview of data structure.

**Aggregate dimensions**	**3rd-order themes**	**2nd-order themes**	**1st-order concepts**
Defining overemployment as desire vs. intention	Quantitative overemployment	Work time length	(1) Reducing contractual and/or actual work time (2) Fit of actual work time to contractual/“normal” hours (3) Length of commuting time (4) Length of holidays (5) Working during “free” time (6) Compensation for long hours
		
		Work time competition (with time outside of work)	(7) Time for family/friends (8) Time for leisure activities (9) Time for recreation (10) Time for personal responsibilities (11) Time for building human capital (12) Time for social commitments
	
	Qualitative overemployment	Work time distribution on tasks	(13) Time for meaningful/important tasks (14) Time for fun vs. boring/routine tasks
		
		Work density	(15) Time pressure (16) Fluctuating workload (17) Working with(out) interruption

Intervening variable	Work time sovereignty	Work time sovereignty	(18) Flexible distribution of time (start, end, breaks) (19) Having a better predictability of time (20) Taking vacation flexibly

Self-reinforcing circle of overemployment	Situational aspects: task demands	Workload	(21) High volume of tasks (22) Unnecessary tasks (23) Lack of personnel resources (24) Low practice/experience with the job
		
		Presence requirements	(25) Presence required for meetings (26) Presence required for business trips (27) Missing out on information when not present
	
	Situational aspects: normative demands	Expectations of others	(28) Expectations of manager/organization (29) Expectations of colleagues/team (30) Customer expectations (31) Expectations in private environment
		
		Deprecation of short hours	(32) Short hours only for an accepted reason (33) Part-time is (un)common within the company (34) Part-time means low career possibilities (35) Problems when switching back from part-time to full-time (36) Part-time is accompanied by unpaid overwork
		
		Appreciation of long hours	(37) Company promotes connection of private and work life (38) Gaining recognition from manager/colleagues by working long (39) Showing presence promotes career success
	
	Personal aspects	Extrinsic motivation	(40) Financial incentives/restrictions (41) Pursuing a career (42) High need for job security
		
		Intrinsic motivation	(43) Being conscientious/meeting one’s own standards (44) Wanting to keep control over one’s tasks/responsibilities (45) Fun at work (46) High motivation to learn

Consequences of overemployment	Psychophysiological strain	Exhaustion/Fatigue	(47) Physical and emotional fatigue
		
		Negative emotions	(48) Feeling stressed (49) Feeling dissatisfied/annoyed
		
		Health impairment	(50) Headaches/backache/others

Step 3: Axial coding and second-order themes. Axial coding (Strauss and Corbin, 1998; Corbin and Strauss, 2008) was used to establish links between the first-order codes and to assemble them under higher-order themes. Step 3 led to 15 second-order categories (see Table 2).

Step 4: Theoretical or selective coding. Finally, the two coders examined the second-order themes with the help of the second author and searched for underlying categories at a higher level of abstraction as well as for connections between higher-level categories. Ideas were discussed multiple times. Seven third-order categories were identified (see Table 2).

## Results

Table 2 illustrates the structure and ordering of the data, from specific first-order categories (staying close to informants’ words) to more general, researcher-induced second-order and third-order themes. Representative quotations that substantiate second-order themes are shown in Appendix B. Within the text, we will give a few sample quotations and write the first-order codes in italics and brackets behind the representative quotations.

The process described above led to four core categories: (1) the definition (facets) of overemployment, (2) causes of overemployment, (3) consequences of overemployment and (4) an intervening variable between overemployment and its consequences.

### Defining Overemployment

#### Desires and Intentions

In our preliminary definition, overemployment is defined as an imbalance between preferred and actual working time where actual working time exceeds preferred time. As overemployment has been defined differently in prior research, we concentrate on this preliminary definition that does not consider financial or workplace constraints. In our interviews, we found people who wished they could work fewer hours, but were prevented from making concrete plans or taking action to reduce their hours by financial or other constraints. However, we also found people who were already planning steps to reduce their work time.

This result can be understood in the context of Perugini and Bagozzi’s (2004) differentiation between desires and intentions. Previous research speaks of preferences, but without explicitly specifying whether preferences refer to desires or intentions. In our interviews we found that it is crucial to differentiate between the two. Thus, we will continue to speak about desires and intentions more specifically. A desire is a “state of mind whereby an agent has a personal motivation to perform an action or to achieve a goal” (Perugini and Bagozzi, 2004, p. 71). Desires strongly influence intentions but are not identical with them. Three aspects determine whether desires are followed by intentions (Malle and Knobe, 1997, 2001): (1) Perceived performability: The perception of an action as performable is influenced by a set of psychological factors, such as self-efficacy, that determine expectations of success. (2) Action-connectedness: Intentions are more strongly linked to goals or outcomes as they imply commitment and at least some form of planning, and (3) Timing: Although both desires and intentions can be now-oriented, future-oriented or refer to an unspecified time, desires are often more time-indefinite, whereas intentions tend to be relatively now-oriented (Perugini and Bagozzi, 2004).

In our interviews, we found people with desires that were not flanked by intentions. Interviewee 24, for example, described having a high workload, as she had different roles to fulfill as a professor (teaching, research, admin tasks, leading a team). She expressed a desire to work fewer hours to reduce the strain she felt she was under. However, when asked if she had any intention of reducing work hours, e.g., by giving up one of her task areas, she answered that she would not want to abandon any of them, for career reasons, but in particular because she liked the combination of her different tasks. Thus, she clearly had a desire to reduce her work hours, but no intention of doing so.

Another example is Interviewee 10, father to a 6-months-old baby. He described a desire to work less and to have more time for his young family. He also said that he would be willing to accept lower pay in general, but not to accept a drop in his current income, as he was the sole earner in his family at the time and was afraid they would not be able to make ends meet if his income were to drop. So he clearly experienced a rather low performability of reducing work hours and his thoughts about reducing work hours were rather time indefinite and thus more characteristic of a desire than an intention:

“It is not like I say, I could reduce 20 percent and it would still be enough. And in a few years when my income will probably be higher, I could better imagine doing this.” (10)

In contrast to this picture, we also interviewed people with the desire to reduce their hours and a clear intention to do so. Interviewee 21, for example, a chef working around 55 h a week, both desired and intended to reduce his work time. He had decided to quit his job in order to switch to an alternative position with fewer hours. So he clearly perceived high performability and high action-connectedness (quitting his job), and his timing was strongly now-oriented.

“Sometimes it is 50, 60, or 70 h, but now I have decided to quit, and I will start in retail.” (21)

“Regarding money I will earn a bit less, but regarding work time it is really good. At some point it was enough, because it simply doesn’t work anymore.” (21)

Interviewee 15, an employee in his 50 s, was also making concrete plans to reduce hours as a form of partial early retirement:

“When I think about it now, I tell myself, when I am 55 at the latest – now I am 52 – I really want to take this step. So, at 55 I want to work less, because I think I can do different things then.” (15)

When asked if this was a concrete plan, he said:

“Yes, definitely. Then in my opinion I don’t have to have the worries that I have talked about earlier, with security and so on.” (15)

Clearly, he had an intention to reduce his work time that was marked by high action-connectedness (concrete plans), a high level of performability (early retirement was available in his company) and a clear plan on when to reduce his hours (timing).

In sum, our examples show that we encountered employees in our sample with either only the desire to cut their hours or with the desire to do so flanked by an intention. This highlights that the definition of overemployment from the subjective viewpoint of employees should focus on desires as the common element. Intentions and feasibility may or may not be given.

#### Quantitative and Qualitative Subtypes of Overemployment

We started with a very general preliminary definition of overemployment. Our interviews have shown, however, that overemployment has more than one facet and demands a more refined conceptualization. Our most important finding regarding the definition of overemployment is the identification of qualitative and quantitative subtypes of overemployment, i.e., overemployment is a multidimensional construct. The quantitative subtype refers to a desire to reduce the absolute time (quantity) people spend at work vs. in other life domains and the time they rather prefer to devote to different areas. The qualitative subtype refers to a mismatch in how time is spent at work (quality) and how people would prefer to spend it. (1) Work time length and (2) work time competition (with time outside of work) are the two dimensions (second-order categories) constituting quantitative overemployment, while (3) work time distribution (on tasks), and (4) work density are the two dimensions constituting qualitative overemployment.

##### Theme 1: work time length

Work time length was coded in all interviews and matched with interviewees’ statements stressing the importance of and dissatisfaction with the length of their working hours. Wishes to reduce contractual and/or actual hours were subsumed under this facet, but so were wishes not to work at times not covered by contracts, e.g., during evenings or weekends. As people perceived commuting time more as work time than as free time, the wish to reduce this was also coded here. Reducing contractual hours and achieving a better fit between actual and contractual work time toward less work time were mentioned equally often:

“A 40-h week would of course be nice, and nothing to do on weekends. This is clearly missing for my full satisfaction with work time.” (*reducing actual work time, working during “free” time*, 3)

“Of course, if I could go home at 4 o’ clock, this would be nice, a part-time job would be ideal.” (*reducing contractual and/or actual work time*, 22)

Another aspect directly connected to work time length was overtime compensation, which was highly valued and desired. Most people valued time compensation over monetary compensation, but it was important to everyone to receive something back for long work hours:

“I think it is important, it is possible at our (company), by contract it is possible, to take leave (…). It is difficult sometimes, because, if you work overtime, then you do it because you have too many tasks. And then you can’t take time off. But I think the possibility to take time off is important.” (*compensation for long hours*, 17)

“I know anyway that it is totally unrealistic, frankly speaking… ok, you work overtime, and the hours, that you really have worked, they are paid, full stop. I think this would contribute substantially to the satisfaction of everyone.” (*compensation for long hours*, 4)

##### Theme 2: work time competition (with time outside work)

Having enough time for things in life other than work was a topic everyone was concerned with. ‘Time for family and friends’ was the topic most mentioned.

“If you have children, then from 9 o’clock in the evening on, it doesn’t matter when you come home, because they are sleeping, and then you cannot say ‘I care for my children,’ because they are already in bed. You come to terms with that.” (*time for family/friends*, 7)

This topic was followed by ‘time for leisure activities’ and ‘time for recreation.’ However, interviewees not only mentioned hedonic activities, but also ‘time for personal responsibilities’ (e.g., moving flat, seeing the doctor), ‘time for building human capital’ (e.g., Ph.D. project or additional self-employment) and ‘time for social commitment’ (e.g., doing voluntary work with refugees):

“I could imagine doing something for refugees. Be it a mentorship, or regularly meeting someone. (…) I could also imagine doing more for old people in the neighborhood, doing their shopping, reading to them, pushing their wheelchairs.” (*time for social commitments*, 7)

Not only was having enough time important for interviewees; they also valued having enough energy left over after work to use time actively:

“You do not have time for yourself. You are in a mill. You work, watch TV, sleep. You do not use your free time. You’re out of power after you’ve worked 9 h.” (*time for recreation*, 7)

##### Theme 3: work time distribution

Work time distribution on tasks encompasses statements that referred to the (wish for a different) distribution of time on work tasks. This facet includes both the desire to spend more time on more meaningful and important tasks (or less time on tasks perceived as unimportant and less meaningful) and the desire to spend more time on fun tasks and less time on routine tasks:

“I would like to have more time to care for our employees and would like to spend less time on unnecessary meetings, discussions and paperwork.” (*time for meaningful/important tasks*, 3)

“I would like to spend less time on meetings. I spend a lot of time in meetings and answering e-mails and I think – both are important – but I think this takes up too much of my work time, it is too large a part, and therefore I have less time for strategic topics or projects that I would like to spend more time on.” (*time for fun vs. boring/routine tasks*, 2)

##### Theme 4: work density

Work density did not refer to a high volume of tasks *per se* (see below: workload), but to the volume of tasks to be completed in a certain time frame. It mainly comprised feelings of time pressure (e.g., having to complete too many tasks in a short time), but also fluctuating workloads over longer time periods (with clear peaks) and the wish to work without being interrupted:

“Work is so tight, because I simply try … to act immediately.” (*time pressure*, 15). “An incoming call - I must act immediately, in the meantime a sales worker stands beside my table and wants me to come by.” (*working with interruption*, 15). “At the same time, an urgent e-mail request comes in. These are just 5 min. And that’s it for about 8–10 h a day.” (*time pressure*, 15)

“It really strongly depends on the time. Now, in the summer, it is of course a bit calmer, but during peaks it is of course significantly more intensive. So it is not an equal flow over the year, but clearly characterized by peaks.” (*fluctuating workload*, 9)

### Causes of Overemployment: A Self-Reinforcing Circle

Our informants reported a variety of aspects which caused and contributed to the quantitative and qualitative subtypes of overemployment. We aggregated the causes to three third-order categories, of which two are situational and one is personal: (a) situational: task demands (“I have to…”), (b) situational: normative demands (“I ought to…”) and (c) personal aspects (“I want to…”) (see Table 2). Our fundamental finding here is that overemployment can never be traced back to a single cause but is the result of and persists because of what we call a *self-reinforcing circle.* This means that situational and personal aspects reinforce each other to cause and preserve overemployment. Before we describe this circle in more detail, we first focus on the themes creating it.

#### Theme 1: “I Have To…”

Task demands were frequently described by the interviewees and could be divided into ‘workload’ and ‘presence requirements’ (Table 2). Regarding workload, interviewees also speculated on the reasons for this high workload, e.g., being understaffed or working on tasks they find superfluous.

“I think jobs are created in such a way that all tasks cannot be done in 40 h.” (*high volume of tasks*, 2)

“I started as HR Manager for Germany. Then I was also responsible for the rest of Europe and my old job was rationalized away. And therefore, my old boss always said, I am my own first clerk, because I don’t have anyone; not because my people aren’t able to do that work, but I just don’t have enough people that I could delegate tasks to.” (*lack of personnel resources*, 19)

“Of course, there are tasks in my job that don’t make sense to me, but they just belong to the job. At the moment, there is this extreme arrangement of meetings, which really binds the energy of a lot of people and the result in the end is only an appointment.” (*unnecessary tasks*, 4)

Interviewees described that it is necessary to show a certain presence, e.g., for meetings, or just to avoid missing out on information.

“A lot of presence is necessary, because I have to be on site, look at things, evaluate them, judge them and talk to people.” (*presence required for business trips*, 20)

“Because it is necessary that you are at the office and don’t do everything from home. You cannot do certain meetings at home.” (*presence required for meetings*, 4)

“If you are not always there to catch everything, you won’t have this information, or only in retrospect and only partially.” (*missing out on information when not present*, 7)

#### Theme 2: “I Ought To…”

Normative demands were the second external source of overemployment. They encompass employees’ description of others’ expectations regarding their work time. Interviewees described people (mostly colleagues or managers) expecting them to work full-time or longer and expecting them to work on certain tasks (distribution aspect) and at a certain pace (density aspect). Norms were communicated directly or indirectly by others, often by criticizing behavior that breached norms. High levels of peer pressure were described, e.g., Interviewee 8 described how others criticized a colleague who went home right after having fulfilled her contractually agreed daily work time, saying “If she goes at half past four, she really can’t be all that busy.” Similarly, Interviewee 17 described colleagues giving her critical looks whenever she goes home without working overtime. Normative demands were also expressed through appreciation of long hours and deprecation of short hours. For example, interviewees described (fearing) worse conditions if they switched to part-time work, mentioning among other details that reducing hours and going part-time meant cutting back on one’s career ambitions and losing interesting tasks, or that it could lead to people continuing to work as much as before, but now on lower pay. Also, in most work environments, short hours were only acceptable for special reasons (e.g., having children):

“[My colleague] works part-time, because she has a small child. But without having children, I do not think anyone would understand if I said I don’t want to work that long, because then she (meaning the boss) would think I am not motivated.” (*short hours only for an accepted reason*, 11)

Long hours, by contrast, were described as highly appreciated and beneficial for employees’ status and careers. Some work environments were also designed to conflate personal and work life:

“The trend was toward blurring the line between personal and work life… small parties took place… there was a fridge with some alcohol… It was officially communicated that the company planned to create something like a living community.” (*company promotes connection of private and work life*, 13)

#### Theme 3: “I Want To…”

Overemployment was partially caused by personal aspects. Interviewees wanted to achieve certain goals and therefore worked in a way that led to overemployment. According to the goals people pursued, we divided personal aspects into extrinsic and intrinsic motivators (Ryan and Deci, 2000). On the extrinsic side, financial incentives were mentioned most often, followed by career opportunities and job security. On the intrinsic side, people described themselves as being conscientious and wanting to meet certain standards. Having fun at work also made them likely to work more than they wanted to. Other intrinsic motivators were the wish to retain control over one’s tasks/areas of responsibility and the motivation to learn, especially when new in a job. Extrinsic motives were mostly mentioned at the very beginning of the interviews, intrinsic motives typically later and when digging deeper, e.g.:

“To be honest I have thought about it” (means reducing work time), “but it always has to do with a financial aspect.” (*financial incentives*, 2) and “But I also want to complete the tasks that I have or that I see as mine. That is also an inner attitude thing.” (*being conscientious/Meeting one’s own standards*, 2)

#### The Circle of Issues Causing Overemployment

In all interviews, overemployment was attributed to more than one issue. Personal aspects and situational demands (normative and/or task demands) always interacted and created a self-reinforcing circle that made it difficult for persons to escape overemployment. An example of how personal aspects and workload interact was given by Interviewee 7, a banking specialist with two grown-up children. She described herself as a conscientious person (personal aspect) leading to a high workload (task demands). She experienced fun (personal aspect) while performing these tasks, and this created overemployment:

“I work without stopping, I am such a working type. In my job there are a lot of people who love chatting, but I do this rarely, because otherwise I don’t get my tasks done.” (*being conscientious/meeting one’s own standards*, 7). “I have created quite a high workload for myself.” (*high volume of tasks*, 7). Sitting here, and the day doesn’t pass by, because I don’t have anything to do, that would be terrible for me. I work on topics because I think they are interesting, or I want to do them.” (*fun at work*, 7). “In comparison to other colleagues I have a full desk. When I work longer, it is not because I dawdle, but because I have to manage the work I have created.” (*high volume of tasks*, 7)

Interviewee 15 provides an example for an interaction of personal aspects with normative demands. He described himself as being toward the end of his career in a company that appreciates long hours, especially for those who wanted to make a career. As he wanted to preserve his career and financial position, he felt he had to stick to the company rules and mores:

“In such a big company as ours, where there is continuous reorganization, you have to repeatedly demonstrate your work in front of the leaders. You must present what you do so that they can make sense of it.” (*showing presence promotes career success*, 15). “If you don’t promote yourself, and I don’t mean showing-off, but simply showing what you do, if you don’t do that, then you fall down career-wise.” (*pursuing a career*, 15)

Then he describes both extrinsic (financial) reasons and intrinsic motivation (control over one’s own tasks) that lead to long working hours:

“It would work to reduce to 30 h, …if we cut down spending. It is this striving for security. Other people get along with much less. It always works with less, I am sure. It is this striving for security.” (*financial incentives*, 15) “I am responsible for certain products and I want to keep this responsibility. If I reduce to 30 h, then someone else takes over and some really nice tasks get lost. I would regret that.” (*wanting to keep control over one’s tasks*, 15)

An example of an interaction between all three themes causing overemployment is provided by Interviewee 8, a woman at the beginning of her career who described a continuously high workload with corresponding expectations from colleagues. She also described herself as conscientious and as wanting to retain control over her tasks; this led her to fulfill others’ expectations and meet high task demands:

“It is continuous high strain, it is not like it calms down a bit from time to time.” (*high volume of tasks*, 8) “And you must always explain yourself, even though you are working overtime, if you go earlier. So, you can never go without a reason, just because the weather is nice, but you must have a reason.” (*expectations of colleagues/team*, 8) “I have a lot of different topics, which is the most interesting part of my job, and I wouldn’t want to hand something over.” (*wanting to keep control over one’s tasks*, 8) “I also explained to my colleagues that I have a bad conscience when I go earlier.” (*being conscientious/meeting one’s own standards*, 8)

Throughout the interviews, it was clear that personal and situational aspects reinforce each other to create overemployment. Although it is difficult to make out the starting point of the circle, the fact that personal aspects were mentioned in all interviews strongly hints at personal aspects being the key to overemployment. This is also reflected in statements made in the interviews, e.g.:

“Actually, no one tells me to work on weekends, but sometimes I put myself under pressure and I do it although no one demands it. And my colleagues do it as well. Therefore, you have to pay attention – you are responsible for yourself – that you use the opportunity which your employer gives you.” (*being conscientious/meeting one’s own standards*, 17)

### Consequences of Overemployment: “It Is Not Stressful Yet” vs. “I’m Dead as a Doornail.”

The variance of described psychophysiological consequences in our data was surprising, given that existing overemployment theories (e.g., P-E-fit theory, Angrave and Charlwood, 2015) would clearly suggest negative psychophysiological consequences. However, six out of 26 interviewees reported no psychophysiological consequences and the remaining interviewees reported levels of strain varying from low to high. We could not make out a significant difference between those reporting desires and those indicating desires and intentions regarding the severity of consequences. However, the level of strain was congruent to the perceived importance of the issue of work time in peoples’ lives. To illustrate this, we highlight examples of low vs. high strain. For Interviewee 9, the importance of work time was relatively lower than that of other job characteristics (e.g., career prospects, financial success). Overemployment in terms of work time was accompanied by mild psychophysiological strain. He worked as an executive assistant and said that he would generally like to work less and at a lower density than currently, but did not see the need to act yet.

“I knew what I was getting into and I think this is very important, and consequently I don’t feel it is too unpleasant to spend so much time here.” and “I accept this, to get ahead in my job […] and now, I feel that it is a reasonable extent, and that I don’t do anything I don’t want or that I am in a hamster wheel where I can’t get out. This feels right at this moment in my life. And if it is getting too much, we have to change it.” (9)

In contrast, other interviewees reported more severe psychophysiological consequences. Most importantly, strong feelings of exhaustion/fatigue were reported, but negative emotions (dissatisfaction/annoyance and stress) and sometimes health consequences also featured:

“There were nice colleagues at the agency. But to know that these are the people I see the longest time during the week, although I would not have chosen them as friends […], was a bit annoying.” (*feeling annoyed*, 13)

“When I come home I’m dead as a doornail” and “you don’t do anything anymore, you don’t feel like doing anything, do you understand? And therefore, you only have, yes, you only have your holidays left.” (*physical and emotional fatigue*, 22)

“I often have a headache if I don’t watch it. I also was in the MRI scanner, but nothing was found, it is more like a tension-based headache.” (*headache*, 15)

### The Role of Work Time Sovereignty

In our interviews, not everyone suffered from psychophysiological consequences. The results indicate that this may partially be explained by a moderating variable, work time sovereignty. Work time sovereignty means having control over when one works (timing of work). This refers to flexible work time regarding daily start and end times and the timing of breaks, as well as to the distribution of working time over longer time periods, e.g., when vacations can be taken. Another aspect of work time sovereignty was predictability and consequently the ability to plan ahead. For those who reported no or only low psychophysiological consequences despite being overemployed, sovereignty was mostly higher than for those reporting stronger psychophysiological consequences. Two examples illustrate this. Interviewee 5 was self-employed and reported no psychophysiological consequences despite being overemployed and preferring less than her current 60 h per week – but she experienced high work time sovereignty:

“Regarding working time, yes, I could imagine reducing a bit, in order to have more possibilities for leisure as well.” and “At the moment it is very flexible […], because in the meantime I am self-employed. […] I can organize my work myself. I can start later in the morning and then in the evening I have an event, where I also invest time.” (*flexible distribution of time*, 5)

In contrast, Interviewee 9 reported overemployment with limited work-time sovereignty and consequently high psychophysiological strain. She used the words “it is an immense strain” and “it massively bothers me” to describe the consequences of overemployment and described her situation as follows:

“It is like that, I have 42 h and I am not very happy with that.” and “At the beginning there were very rigid work time rules, a fixed starting time and a fixed ending time. Recently, it has become a bit looser, so now I have a fixed core time, which, however, also has a wide range, so I don’t get away in under 8 h.” (*flexible distribution of time*, 9)

In general, sovereignty was seen very positively and directly influenced satisfaction, with work time sovereignty lowering psychophysiological consequences:

“Good work time for me definitely always contains flexibility.” (*flexible distribution of time*, 4)

“I can always say, if the weather is nice, I go to the playground with my child and stay longer in the evening or stay longer the next day or so, flexibility is the main thing.” (*flexible distribution of time*, 25)

### A Grounded Theory of Overemployment

**FIGURE 1:**
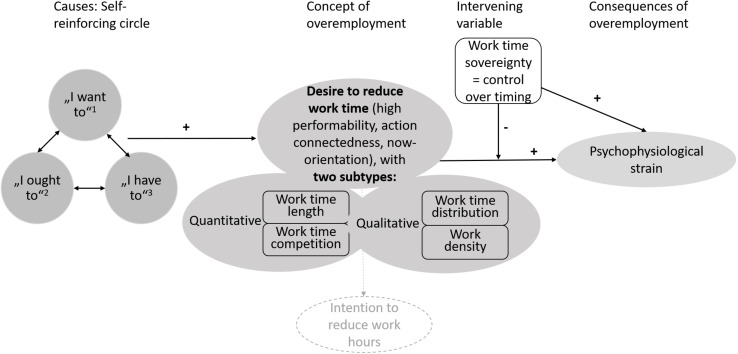
A theory of overemployment, its causes and its consequences. Superscript numbers refer to coded themes: ^1^personal aspects, ^2^normative demands, and ^3^task demands. The dashed line means intentions may or may not follow from desires.

This article has attempted to contribute to understanding the concept, causes and consequences of overemployment from the employee perspective. Figure 1 integrates all our findings into an overall framework.

The following propositions are set forth on the basis of the description above:

#### Proposition 1

Overemployment is a desire to reduce work time (either overall, on certain tasks, or in a particular time period). It is reflected in two subtypes: (a) a quantitative mismatch of work time with time outside of work, i.e., work time length and work time competition (with time outside work), and (b) a qualitative mismatch of time at work, i.e., work time distribution (on tasks) and work density.

#### Proposition 2

Overemployment is caused by a self-reinforcing circle of personal needs and situational (task and/or normative) demands.

#### Proposition 3

Overemployment may have negative psychophysiological consequences, i.e., exhaustion, negative emotions or impaired health.

#### Proposition 4

The positive relationship between overemployment and its psychophysiological consequences is moderated by work time sovereignty: higher levels of work time sovereignty buffer the negative effect of overemployment on psychophysiological consequences. In addition, work time sovereignty has a positive direct effect lowering psychophysiological strain.

## Discussion

### Theoretical Implications

Our data analysis has led us to construct a theoretical framework that can be related to existing literature (e.g., Reynolds, 2003; Golden and Gebreselassie, 2007), yet also expands and refines it. Using the principles of Grounded Theory and Thematic Analysis, we have developed codes systematically and worked out the facets underlying the phenomenon of overemployment (Boyatzis, 1998). Regarding the conceptualization of overemployment, we found that it is important to focus on desires over intentions. A desire represents the wish of an employee to work fewer hours. Desires are believed to influence future outcomes, including the intention to reduce work hours. Although Fishbein and Stasson (1990) believe that intentions are motivational in nature, Bagozzi (1992) argues that desires are distinct from intentions and asserts that intentions may not be activated unless desires are present. For this reason, we propose that desires for fewer work hours will positively influence employee intentions. In our sample, we found people describing themselves as overemployed who currently desire fewer hours and intend to reduce their working time. But we also found people facing varied constraints who desired to reduce their hours but had no intention of doing so. The common element here, however, was a desire for fewer work hours.

Our main finding is that overemployment is a multi-faceted construct. Most prior research has measured only the length of time worked before classifying individuals as “matched,” or “overemployed” (e.g., Bielenski and Wagner, 2003). However, this simplified conceptualization has proved problematic, as studies using only slightly different items have shown strongly divergent rates of overemployment (e.g., Holst and Bringmann, 2016) and research participants have found it difficult to indicate exact working time desires with precision (Campbell and van Wanrooy, 2013). Our theoretical framework takes this into account: we define overemployment as a desire (according to the desire definition in Perugini and Bagozzi, 2004) to reduce work time (either overall, on certain tasks, or in a particular time period). Overemployment refers in one or more ways relating to the length of time worked, time competition, work density and work time distribution.

Overemployment is caused by a combination of personal needs and external factors (normative and/or task demands) reinforcing each other, and this reinforcement may contribute to its persistence. Prior literature has focused on individual and mainly external aspects in the development of overemployment, especially on normative pressures (e.g., Landers et al., 1996; Eastman, 1998), task/work characteristics (van Echtelt et al., 2006; Matta, 2015), occupational and industry characteristics (Golden and Gebreselassie, 2007) or demographic characteristics (e.g., Reynolds, 2003; Golden and Gebreselassie, 2007). Our interviews show that individual motivation together with situational aspects may contribute to a better explanation of overemployment. Reynolds and Aletraris (2010) found that mismatches persist for extensive periods of time (i.e., 5 years, in their study). The dynamics of the circle may be one explanation for this persistence.

Our theoretical framework has also highlighted the distinction which can be made between overemployment and the psychophysiological consequences of overemployment. This is in line with previous research showing that working more than preferred correlates with lower job satisfaction (Wooden et al., 2009; Wunder and Heineck, 2013; Angrave and Charlwood, 2015), poorer health (Bell et al., 2011) and lower life satisfaction (Wooden et al., 2009; Angrave and Charlwood, 2015). However, not all employees are equally affected. According to our theory, the relationship between overemployment and its consequences is moderated by work time sovereignty. The influence of this moderator may also explain the inconsistent prior results relating to the impact of overemployment on life satisfaction (e.g., Friedland and Price, 2003; Wunder and Heineck, 2013 vs. Wooden et al., 2009; Angrave and Charlwood, 2015). The effects of the moderator are in line with research findings on the positive effects of schedule control on job satisfaction (e.g., Krausz et al., 2000).

Our theoretical framework proposes an integrative approach to overemployment that may prove very useful for work time literature in general, especially as overemployment is widespread among employees, whose own voices have nevertheless only seldom been analyzed in detail. Finally, the propositions we have derived in our qualitative study may also serve as a basis to generate hypotheses to be tested in a quantitative study – also with larger, representative samples (Boyatzis, 1998).

### Practical Implications

Our multi-faceted theory of overemployment can serve as a basis for developing a new measure of overemployment that encompasses all four facets of overemployment and could lend itself to mapping overemployment within individual companies, comparing different teams or departments, and generating results that could form the basis for targeted healthcare initiatives or employee training measures. Describing overemployment as a multidimensional construct is also helpful when it comes to acting to combat it. It is clear now, for example, that reducing working hours by moving to part-time work may not always represent the best way to reduce overemployment, since improvements in work time length could come at the cost of increased work density. The reorganization of tasks, however, may help to reduce work density (or positively modify work time distribution) and therefore also reduce overemployment. People who have more fun at work and are under less time pressure might also prefer to work longer. If part-time positions are introduced, but jobs are not adequately redesigned, work time distribution could worsen, since part-time work often includes fewer challenging tasks. Before planning a course of action, it therefore makes sense to take a holistic view and to look at the complete picture of overemployment.

As was also apparent in our interviews, work time is often a topic companies choose to ignore, since reducing (unpaid) actual working hours means higher labor costs. However, not broaching the topic may lead to dissatisfaction and employee health problems that also impact negatively on companies, for example through greater rates of absenteeism and employee fluctuation. The identified causes of overemployment and intervening variables already point to strategies for reducing overemployment or minimizing its negative consequences. Some strategies may come at a high cost to companies, e.g., employing more people to reduce task demands, while others may come at a low cost or, indeed, cost little or nothing, e.g., improving work processes or facilitating time models like job sharing or working from home. Reducing normative demands may be a bigger challenge for employers, since organizational cultures typically evolve gradually over time and are resistant to change (Schein, 1990). Supervisors could play a major role here, because they may or may not support employee work time priorities and serve as good role models.

Our interviews show that enhancing work time sovereignty is crucial to reducing the negative emotional consequences of overemployment. Flexible working hours and moving toward results-only work environments may represent a possible solution to increasing work time sovereignty (Ressler and Thompson, 2008). However, some regulation still seems to be necessary, as other results (Matta, 2015) show that unregulated work hours can lead to higher overemployment.

### Limitations and Directions for Future Research

The results of our study must naturally be viewed considering some limitations. Although it is not a necessary step in conducting Grounded Theory (Corbin and Strauss, 1990, 2008; Charmaz, 2014), comparing people who perceive themselves as overemployed and people who do not might usefully have served to further explore the causes of overemployment by making comparisons between both groups.

In addition, this study did not look at a representative sample of the German workforce, or the workforce in any other country. Our sample consisted of highly educated, well-paid employees. Thus, none of them suffered from economic hardship; which is probably not the case for all overemployed persons. Future research should therefore seek to validate our theoretical framework for a larger and more diverse workforce. It would be interesting to explore overemployment as it affects employees with lower levels of educational attainment and lower incomes. Research indicates that people from poorer backgrounds face greater family demands. Together with their lower resources, this leads to less time for work (Pitesa and Pillutla, 2019). Competing work and family demands may therefore be a crucial component in poorer workers’ overemployment.

Within the European context, German working culture is characterized by medium flexibility and a strongly regulated labor law environment (Eurofound, 2016, 2017). It may be asked whether and in how far our results are transferable to other countries with different working time cultures and legal regulations. Additionally, research has shown that people typically overestimate their weekly work hours when asked to estimate them in retrospect (Robinson et al., 2011). Overestimation may have occurred here, as only about half of the interviewees documented their work hours on a daily basis, while others reported their estimated weekly work hours. However, as we focus on subjective experiences here, this may be a minor problem. Another possible limitation relates to the strong focus of our theory on the employee perspective. An organizational perspective giving more attention to, say, opinions held by HR management experts or leaders could add an extra dimension to our results, as managers might, for example, have different insights into the causes of overemployment in their organizations. Future research should consider the organizational perspective, especially in relation to the development of strategies for combating overemployment. Our theory also needs to be further tested with different samples quantitatively and qualitatively. Regarding quantitative research we strongly suggest developing a scale on overemployment based on our findings of the overemployment concept. Different from the past one-item measures a scale could map the four different dimensions of overemployment, and also could differentiate between desires and intentions. In larger quantitative studies it would also be interesting to examine whether people with particular subtypes of overemployment differ, e.g., on whether they have intentions to change their situation or which consequences of overemployment they experience (e.g., consequences for well-being, but also performance or turnover). Although we did not find that people with desires versus those with desires and intentions to reduce work time differed regarding psychophysiological strain, this could as well be tested in larger quantitative studies using an overemployment scale. Our theory should not be seen as complete, but as open to enhancement, as there may be other consequences, e.g., in relation to turnover or performance, that we did not identify in our data. Although we found initial indications that work time sovereignty acts as a moderator, this needs to be tested in a quantitative study with a larger sample in the future. Additional moderators may yet be discovered between overemployment and consequences e.g., social support. Our theoretical framework is also rather static. Reynolds and Aletraris (2006) showed a dynamic picture of hour mismatches as they are created and resolved within the context of a fluid labor market. Using longitudinal data to track changes in the levels of overemployment people encounter over their working lives might aid understanding of the causes of overemployment.

Given the limitations described, the ideas presented need to be tested in future quantitative studies. The conceptual model presented here may help to inspire and guide fresh research.

## Data Availability

The raw data supporting the conclusions of this manuscript will be made available by the authors, without undue reservation, to any qualified researcher.

## Ethics Statement

This study was carried out in accordance with the APA ethical guidelines. Participants were informed about the study’s aims and duration and about how to obtain information on the results. They were also informed about whom to contact with any questions regarding the research and about their right to leave the survey and interview at any time. Subjects were guaranteed anonymity and confidentiality of their data. The Ethics Committee of the University of Bamberg has reviewed and approved the study.

## Author Contributions

JH and MA were responsible for the research conception, and iteratively revised the first version of the manuscript and developed it further. JH was responsible for the recruitment of participants, and conducting the interviews and the first round of coding and analysis, and wrote the first version of the manuscript.

## Conflict of Interest Statement

The authors declare that the research was conducted in the absence of any commercial or financial relationships that could be construed as a potential conflict of interest.

The handling Editor declared a shared affiliation, though no other collaboration, with the authors JH and MA.

## Footnotes

1 Numbers in brackets indicate the number of the interviewee being cited (see Table 1).
