# Supplementary material for: “Because Work Time Is Life Time” – Employees’ Perceptions of Individual Overemployment, Its Causes and Its Consequences
Source: Front Psychol. 2019 Aug 16;10:1920. doi: 10.3389/fpsyg.2019.01920 (PMC6707378; doi:10.3389/fpsyg.2019.01920)
Supplement: Supplementary file 1 [file Data_Sheet_1.PDF]

Appendices

Appendix A

Interview protocol (key questions) for overemployment interviews.[1]

I want to find out more about people who prefer working fewer hours than they currently work. Please tell me a bit about your current work and work time situation?

What does your work time currently look like? Please describe it for me!

- Tell me about the length of your work time!
- When do you work?

If you consider your current work time, how would you describe your satisfaction with your work time?

- With what aspects are you satisfied?
- With what aspects are you not satisfied?
- Did you have different working hours in the past?
  - If so: What was positive/negative about that change? What changed for you?
  - If not: Next question
- Where do you see the advantages and disadvantages of your current work time?

How do you feel about your work time?

In to your opinion, what makes for good or bad working time?

- How important are the aspects you mentioned (as good or bad for work time) for you?
- For important aspects: Why is this important?
- For unimportant aspects: Why is this not important?

What would your ideal working time look like?

- What would it look like considering financial aspects?
- And not considering financial aspects?
- Which other aspects are important to you besides financial aspects when thinking about (reducing) work time?
- How important are these aspects to you, and why?

What makes your work time exceed or differ from your preferences?
If you reduced your work time, how would your environment react?
In your opinion, who is responsible for good work time?
How could your ideal work time be achieved?
We have talked about work time and preferences for quite a while now. Is there anything else I should know to understand this topic better? Would you like to add anything?

[1] Questions were adapted according to the course of the interview. They don't reflect all questions asked, nor were all questions asked always. Sometimes the order of questions was also varied to better follow the interviewee.

Appendix B

Representative quotes (in vivo codes in bold), first-order themes (in brackets) and second-order themes

**Work time length**
However, I know from my colleagues that they are also **taking their work home**. I haven't done this so far, because **weekends are weekends**. And I try to stick to that. (working during "free" time, 8)<br>Because I must consider these **long commuting times**, I don't come out, if I want an interesting job (commuting time), it is like that, and therefore, I would reduce to a **four-day week.** This is my wish. **Four days** to have the Friday off. (reducing contractual and actual work time, 22)
**Work time competition**
I have a lot of **hobbies,** which are neglected a bit now. I **read a lot. I go hiking. I play golf**. (time for leisure activities, 7)<br>Because I thought, I could achieve it, to do a **PhD next to a 40-hour week**, and then I realized that it is not doable for me. I mean, the **PhD** that I do at the same time. (time for building human capital, 13)
**Work time distribution**
There are **tasks I like more** and it would be nice to have **more time for those tasks**. (more time for fun tasks, 14)<br>I would like to invest even **more time to reorganize the working space**, to better organize it. (more time for important tasks, 4)
**Work density**
From Monday to Thursday working hours are from 9 to 5 or maybe 6 on average. And then **on Friday it goes up promptly**, so that you start at 9 and really work till 10, 11 or 12. (fluctuating workload, 21)<br>But it is **not like you ever have a period**, where you can say "Ok, now I work **a bit slower**." I have never experienced that since I am at the company. That means at times it is **even more stressful**. (time pressure, 8)
**Work time sovereignty**
And for my employer it would be important to cancel these **limitations** (of presence) **in the morning and evening.** (flexible distribution of time, 9)<br>**Flexible work time** is one point. For every doctor's appointment or any other thing, that changes my work time, I **must ask** if it is ok. And I think this is exhausting. (flexible distribution of time, 23)
**Workload**
And when I took over the department lead 5 years ago, where it was clear, ok, now the **workload is getting even higher** again. (high amount of tasks in general, 6)<br>And the other thing is that I had **difficulties at the beginning**, evaluating which topics I can **delegate** to my employees, because I didn't know them too well, and then the risk is – especially under time pressure – that I **do certain topics on my own**, that maybe an employee could do pretty good as well. (low practice/experience, 6)
**Presence requirements**
You also **work internationally with other countries** and they have different time zones. Therefore, you are **restricted** again. For example, Asia and the US, they are not in our time zone, and then you must see how you can catch them. (presence required for meetings, 12)<br>Now in January it wasn't possible (to work less), and the reason was, there was a lot of **business travelling**, where **more hours** came up. (presence required for business trips, 15)

**Expectations of others**
In principl**e, it is done when the customer is done.** (Customer expectations, 21)<br>I don’t think that this (= request for working less) would necessarily be rejected, but I think **my boss** firstly **would try to prevent me** from doing this. (expectations of manager, 9)<br>I think that I would be **teased by my friends** that I am a lazy dog. (*explanation by the authors:* means when reducing work time). (expectations in private environment, 16)
**Depreciation of short hours**
I don’t know what the possibilities are, I have heard that it is **difficult to switch back to full-time**. (problems with switching from full-time to part-time, 14)<br>Or I want to **reduce** my work time, for example to have the Friday or Monday off, but therefore I **pack so much into the other days**, that I am actually **back at my old work time**. (part-time is accompanied by unpaid overwork, 19)
**Appreciation of long hours**
But I think that someone, who is prepared to **sacrifice himself**, and to **put himself at the end of the queue**, has better **chances to be promoted.** (presence promotes career success, 18)<br>Because one still hasn’t said completely goodbye to the **presence culture**. I think, it is still there. (presence promotes career success, 19)
**Extrinsic motivation**
And of course, I realize, that regarding the **financial** situation, you have certain freedoms, which of course is great. (financial incentives, 6)<br>This(position) had offered me a **career possibility** back then, and I took it. Regarding work time, it would have been nice if I had reduced. (pursuing a career, 15)
**Intrinsic motivation**
I think, this is also the phase where you say you will work more and really **want to start right away**, you have an **interesting team** and you **learn a lot.** (high motivation to learn, 8)<br>Sometimes there are these situations where you gain the impression, oh, there are others who could **compete a bit with me for my position** […] And then of course I am happy that I am not a part-time worker, because then I couldn’t **fight for my position** quite as well. (keep control over tasks/responsibilities, 15)
**Exhaustion/Fatigue**
(I wish) that I was not so **exhausted**, this **permanent fatigue**. (exhaustion, 12)<br>I am glad if I still get to see the evening news. If I sit on the couch, I might as well go to bed, because **I can’t keep my eyes open**. (exhaustion, 22)
**Negative emotions**
It really **feels like too much**, because it simply is too much. (feeling stressed, 25)<br>(You) feel a bit like a **hamster in a wheel**. You just don’t make it. (feeling stressed, 8)
**Health impairment**
Often there were situations, where I suddenly got **sick, really sick**. And my doctor said: ‚but haven’t you noticed that before?’ (other health related issues, 15)<br>If I **listen to my body**, it would sometimes say ‘Hey man, one or two hours less per day on average would be good for you.’ (other health related issues, 8)

Appendix C

Example for steps of the analysis

Step 1. Open coding: Using in vivo codes helped the researchers stay close to the data and insured the likelihood that the different coders encode the same information similarly (Boyatzis, 1998). For example, one interviewee indicated that he would want to “basically work less” (9) and another mentioned that “pressure is high” (26), which both coders coded as in vivo. During this step hundreds of in vivo codes were generated which were then analyzed in Step 2.

Step 2: First-order categories: To give an example, the coders found that informants described various perceived disadvantages and obstacles of working part-time as a means to reduce work hours, e.g. the fact that part-time work is uncommon, cannot be realized without having an accepted reason (e.g. having children), or leads to challenges when switching back to full-time. The coders factored these aspects into the category “deprecation of short hours”, as it described what these aspects had in common.

Step 3: Axial coding and second-order themes: Continuing with the example, the coders identified that “deprecation of short hours” together with other first-order categories, i.e. “appreciation of long hours” and “expectations of others”, indicated normative demands that were imposed on the individual and contributed to overemployment.

Step 4: Theoretical or selective coding: Two of the third order codes referred to facets of overemployment as perceived by the overemployed interviewees (“quantitative overemployment” and “qualitative overemployment”) and three referred to perceived causes of overemployment (“task demands”, “normative demands”, “personal aspects”), which together formed a self-reinforcing circle preserving overemployment. Another category referred to overemployment having negative psychophysiological consequences, and a final one referred to work time sovereignty.
